# *Aerococcus urinae* — significance of detection in the paediatric urinary tract: a case series

**DOI:** 10.1007/s00431-022-04730-2

**Published:** 2022-12-06

**Authors:** Dimitri Rast, Katrina Suzanne Evers, Adrian Egli, Christoph Rudin, Alexandra Goischke, Nicole Ritz

**Affiliations:** 1 University Children’s Hospital Basel UKBB, Spitalstrasse 33, 4056 Basel, Switzerland; 2 Paediatric Nephrology, University Children’s Hospital Basel, Basel, Switzerland; 3 Division of Clinical Bacteriology and Mycology, University Hospital Basel, Basel, Switzerland; 4 Applied Microbiology Research, Department of Biomedicine, University of Basel, Basel, Switzerland; 5 Paediatric Infectious Diseases and Vaccinology, University Children’s Hospital Basel, Basel, Switzerland; 6 Department of Paediatrics and Paediatric Infectious Diseases, Children’s Hospital, Lucerne Cantonal Hospital, Lucerne, Switzerland; 7 Department of Paediatrics, The Royal Children’s Hospital Melbourne, The University of Melbourne, Melbourne, Australia; 8 Institute of Medical Microbiology, University of Zurich, Zurich, Switzerland

**Keywords:** Urinary tract infection, Pyelonephritis, Malodorous, Children, Urogenital disorder, CAKUT

## Abstract

*Aerococcus urinae (A. urinae)* is primarily recognized as a common pathogen in the geriatric population, causing urinary tract infection (UTI), sepsis, and endocarditis, predominantly in female patients. In the paediatric population, only a few case reports exist suggesting *A. urinae* causes malodorous urine in otherwise healthy boys. In this study, we investigated the spectrum of clinical and laboratory presentations of *A. urinae* detection in children. A retrospective, single-centre, case series including all patients with the detection of *A. urinae* during a 7**-**year study period. Patients with detection of *A. urinae* only in non-urogenital skin swabs were excluded. A total of 40 samples from 33 patients were identified of which 20 patients were included in the final analysis. The median (IQR) age was 6.8 (2.9–9.5) years; 18 (90%) patients were boys. Four patients were diagnosed with a UTI, six had malodorous urine without UTI, three were diagnosed with balanitis and seven showed *A. urinae* colonization in the urine culture. Urogenital disorders were present in 12 patients. Additional pathogens were detected in 13 patients. Recurrence of detection during our study period was observed in four (20%) patients.

*  Conclusion*: Beyond malodorous urine, *A. urinae* detection is associated with more severe presentations including UTI in the paediatric population. Pre-existing urogenital disorders were frequent, and therefore, a nephro-urological investigation should be considered in all cases of *A. urinae* detection in the paediatric population.

**Table:** 

**What is Known:** *• Aerococcus urinae (A. urinae) is known to be a common pathogen in the geriatric population, causing urinary tract infection (UTI), sepsis, and endocarditis, predominantly in female patients.* *• In the paediatric population, A. urinae is mainly described as a low-grade pathogen. Some case reports describe A. urinae as the cause of extraordinary malodorous urine in otherwise healthy boys. *	
**What is New:** *• Beyond malodorous urine, A. urinae detection is associated with more severe presentations including UTI in the paediatric population.* *• A. urinae was mainly detected in boys with pre-existing urogenital disorders; therefore, a nephro-urological investigation should be considered in cases of A. urinae detection in the paediatric population.*

**What is Known:**

*• Aerococcus urinae (A. urinae) is known to be a common pathogen in the geriatric population, causing urinary tract infection (UTI), sepsis, and endocarditis, predominantly in female patients.*

*• In the paediatric population, A. urinae is mainly described as a low-grade pathogen. Some case reports describe A. urinae as the cause of extraordinary malodorous urine in otherwise healthy boys. *

**What is New:**

*• Beyond malodorous urine, A. urinae detection is associated with more severe presentations including UTI in the paediatric population.*

*• A. urinae was mainly detected in boys with pre-existing urogenital disorders; therefore, a nephro-urological investigation should be considered in cases of A. urinae detection in the paediatric population.*

## Introduction

*Aerococcus urinae* is a Gram-positive, alpha-haemolytic and catalase-negative bacterium first described in 1953 [1]. The detection has been challenging due to morphotype similarities with streptococci and coagulase-negative staphylococci. Since the introduction of matrix-assisted laser desorption ionization time of flight mass spectrometry (MALDI-TOF MS), *A. urinae* is increasingly detected and reported in the literature [2].

Previously thought to be a contaminant, *A. urinae* is now primarily recognized as a pathogen in the geriatric population, causing urinary tract infection (UTI), sepsis, and endocarditis [3]. Predisposing factors for colonization and UTI with *A. urinae* are as follows: age above 65 years, female sex, underlying systemic medical conditions (e.g. diabetes mellitus, heart disease), and urogenital disorders [4].

It has been suggested, that *A. urinae* is a rare cause of extraordinary malodorous urine in otherwise healthy boys [5, 6]. This is in contrast to the fact that generally, parental reporting of “smelly urine” is not indicative of a UTI [7]. In the case of *A. urinae*, the presence of additional pathogens in urine culture is reported potentially enhancing the extraordinary odour [6]. However, monoculture has been more frequently reported in case reports when *A. urinae* is detected with malodorous urine [5, 8, 9]. For *A. urinae* infection, most case reports suggest it is a low-grade pathogen, with only a few cases of more severe presentations. These include the case of a 12-year-old boy with an acute pyelonephritis [10]. Two further cases (in an 11-year-old and a 17-year-old boy) had a subacute infective endocarditis, both with underlying congenital heart disease and an initial history of malodorous urine [11, 12].

Only little is known about the risk factors and the spectrum of clinical and laboratory presentation of *A. urinae* in children, particularly in those with more severe presentations.

We therefore aim to describe the spectrum of clinical and laboratory presentation of all retrospectively identified cases with the detection of *A. urinae*.

## Methods

### Study design and setting

The study is a retrospective case series including patients identified using the laboratory records of the division of clinical bacteriology at the University Hospital of Basel between January 1st, 2014, and December 31st, 2020. Inclusion criteria were the isolation of *A. urinae* in any sample (urine, blood or swab) of patients cared for at the University Children’s Hospital in Basel, Switzerland and with parental consent to participate in research. Exclusion criteria were the detection of *A. urinae* in non-urogenital skin swabs only and/or missing consent to participate. The study was approved by the ethics commission of Northwestern and Central Switzerland (EKNZ 2021–00321) and was done in accordance with the tenets of the Declaration of Helsinki and in compliance with Swiss patient data protection regulations.

### Microbiology

Microbiological growth was assessed by inoculating 1 µL of urine stabilized by boric acid (Sarstedt urotubes) on a 5% sheep blood agar and non-selective chromagar plate (bioMérieux, Lyon, France). We determined the bacterial species from single bacterial isolates using MALDI-TOF MS (Bruker, Bremen, Germany) using the mass-spectrum library and the MALDI Biotyper 3 software (OC 3.1. Bruker Daltonics) at standard settings. Subsequent antibiotic resistance testing was conducted according to EUCAST recommendations. According to the local resistance situation, all *A. urinae* strains were considered susceptible to amoxicillin. Antibiotic resistance testing was therefore only done on request.

### Data sources

Electronic medical records were used to extract the following data: baseline epidemiological characteristics, clinical presentation, laboratory measurements including bacterial cultures, imaging, treatment and clinical outcome. A database was used for the storage of encoded data extracted from the electronic medical records.

### Data analysis and statistical methods

Stratified analysis was performed in the following groups: UTI, malodorous urine without UTI, balanitis, and colonization. Criteria for UTI were used according to the Swiss Consensus Recommendations [13]. These include clinical signs and symptoms (fever, pollakiuria, dysuria, and loin tenderness) and significant growth of a single uropathogen (≥ 100,000 CFU/ml in midstream urine samples with pyuria and symptoms, ≥ 10,000 CFU/ml obtained through catheterization). Exception for distinct clinical symptoms was made in Neurogenic Bladder, where UTI was diagnosed with none or mild symptoms if significant growth of a single uropathogen and pyuria was present. Diagnosis of acute focal bacterial nephritis (AFBN) was based on magnetic resonance imaging. It is described, that AFBN may occur without significant growth of a uropathogen and with normal urinalysis [14]. Cases without clinical signs of UTI, normal urine analysis and a non-significant bacterial growth with a single dominating uropathogen were classified as colonization. The group “malodorous urine” was differentiated from colonization if an extremely unpleasant smell was reported with a high level of distress in the affected children. Significant pyuria was defined using the current cut-off from the laboratory of the University Hospital Basel (WBC > 20/µl, from July 1st, 2020, WBC > 56/µl after a change of flow cytometry devices). The software used for descriptive statistics was IBM SPSS Statistics (Version 24.0.0.0).

## Results

### Study population

**Fig. 1:**
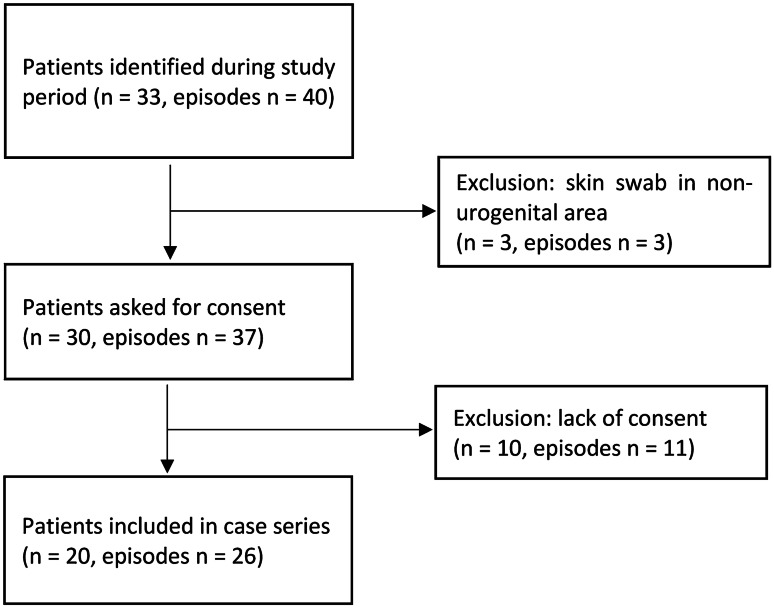
Number of patients/*A. urinae*–associated episodes included in/excluded from the study and reasons of exclusion

During the 7-year study period, *A. urinae* was isolated from 40 samples in 33 patients. A total of 13 patients were excluded from further analysis for the following reasons: parents not available for consent (*n* = 8), consent refused (*n* = 2) or *A. urinae* detection in a non-urogenital skin swab (*n* = 3). Hence, we included 20 patients in the final analysis (Fig. 1). The median (IQR) age was 6.8 (2.9–9.5) years; 18 (90%) patients were boys (Table 1). *A. urinae* was detected in 17 urine cultures and 3 urogenital swabs but not in blood cultures. The final diagnoses were as follows: UTI (*n* = 4), of which two fulfilled the criteria of an AFBN; malodorous urine without UTI (*n* = 6), balanitis (*n* = 3) and colonization without UTI (*n* = 7). The age in the four groups was comparable; however, patients with UTI and malodorous urine tended to be older than patients with balanitis or colonization (Table 1).

**Table 1:** Summary of the baseline characteristics

Characteristics	All patients	UTI	Malodorous	Balanitis	Colonization
Total included, *n* (%)	20 (100)	4 (20)	6 (30)	3 (15)	7 (35)
Age, median (IQR), y	6.8 (2.5–9.5)	5.8 (2.9–10.1)	9.1 (5.7–10.0)	2.9 (-)	2.4 (0.9–9.5)
Male, *n* (%)	18 (90)	4 (100)	6 (100)	3 (100)	5 (71.4)
Urogenital disorders, *n* (%)	12 (60)	4 (100)	4 (66.7)	0 (0)	4 (57.1)
Number admitted, *n* (%)	5 (25)	2 (50)	0 (0)	1 (33.3)	2 (28.6)
Other pathogen detection, *n* (%)	13 (65)	0 (0)	5 (83.3)	3 (100)	5 (71.4)

*UTI* urinary tract infection, *IQR* interquartile range, *y* years

### Clinical findings

The most common reason for the presentation was malodorous urine (*n* = 5, 25%). Further reasons included the following: balanitis (*n* = 3), fever (*n* = 3), abdominal pain (*n* = 2), incontinence (*n* = 2) and acute urinary retention, recurrent UTI and testicular pain (one each). Fever at presentation and during admission/follow-up was reported in five patients, of which three were diagnosed with an *A. urinae* UTI and two had a fever without laboratory proof of UTI. Both patients with AFBN presented with fever and abdominal pain; one patient also complained about more specific one-sided flank pain.

### Urogenital disorders

Urogenital disorders were present in 12/20 patients (60%). Nine patients had an underlying congenital anomaly of the kidney and urinary tract (CAKUT), including the following with several options possible per patient: posterior urethral valves (*n* = 5), epispadias (*n* = 2) and hypospadias (*n* = 2), and one each with hydronephrosis, vesicoureteral reflux (VUR), urethral diverticulum, bladder diverticulum, urethral fistula and bladder exstrophy. Further urogenital disorders included the following: bladder bowel dysfunction (*n* = 3), neurogenic bladder because of myelomeningocele (*n* = 2) and phimosis (*n* = 1) (Table 2). In patients with urogenital disorders, 4/12 (33.3%) had repeated *A. urinae* detection in urine cultures, whereas in patients without an underlying disorder, none had repeated *A. urinae* detection.

**Table 2:** Detailed findings of all included patients

	**Clinical characteristics**			**Urogenital disorders**									**Laboratory findings**				**Treatment**		**Outcome**
				**CAKUT**						**Others**									
**No.**	Age (y)	Sex (m/f)	Group	PUV	Birth defect urethra	Bladder diverticulum	Bladder exstrophy	Hydro-nephrosis	VUR	BBD	Neurogenic bladder	Phimosis	*A. urinae* (/ml)	Other pathogen detection	WBC count in urine (/µl)	CRP max (mg/l)	Antibiotics used	Hospital admission	Recurrent detection
1	10.9	m	UTI with AFBN							x		x	10^4		32.3	98.6	Ceftriaxone, Cefpodoxime	x	
2	3.7	m	UTI with AFBN	x		x							10^6		19	268.4	Ceftriaxone	x	
3	2.6	m	UTI						x		x		10^6		3179.5		AMC		x
4	7.9	m	UTI		x			x					10^6		85		Cefpodoxime, AMC		x
5	10.0	m	Malodorous	x									10^5		2.7		AMC		
6	9.5	m	Malodorous							x			10^6	x	-		AMC		
7	5.7	m	Malodorous	x						x			10^5	x	93.6		AMC		
8	8.2	m	Malodorous	x		x							10^6	x	14.9		AMC		x
9	8.7	m	Malodorous										10^4	x	-		AMC		
10	10.1	m	Malodorous										10^5	x	88.8		AMX		
11	4.3	m	Balanitis										ns	x	-				
12	2.9	m	Balanitis										ns	x	-				
13	1.6	m	Balanitis										ns	x	-	13	AMC, Ceftazidime	x	
14	0.9	f	Colonization								x		10^4	x	87.5				
15	9.5	f	Colonization		x								10^4	x	11.9				
16	8.2	m	Colonization		x		x						10^5	x	22.2				x
17	12.7	m	Colonization	x	x								10^4		317				
18	1..1	m	Colonization										10^3		12.4	6	Cefpodoxime	x	
19	2.4	m	Colonization										10^4	x	29	190	Ceftriaxone	x	
20	1.1	m	Colonization										10^3	x	98.5	< 5	Cefpodoxime		

*AFBN* acute focal bacterial nephritis, *AMC* amoxicillin-clavulanic acid, *AMX* amoxicillin, *BBD* bladder bowel dysfunction, *CAKUT* congenital anomalies of the kidney and urinary tract, *CRP C*-reactive protein, *ns* not specified, *PUV* posterior urethral valves, *UTI* urinary tract infection, *WBC* white blood cell

### Microbiology

*A. urinae* was detected in 17/20 cases (85%) in urine cultures and in 3/20 cases (15%) in urogenital swabs. Of those patients with *A. urinae* detected in urine cultures, 7 were monocultures and 10 (all non-UTI) had other potential pathogens identified: including *Escherichia coli*, *Klebsiella oxytoca*, *Enterococcus* species, *Facklamia* species and *Staphylococcus epidermidis* in urine. In urogenital skin swabs, none was a monoculture for *A. urinae* and further potential pathogens were identified including *Streptococcus anginosus*, *Enterococcus faecalis*, *Proteus vulgaris*, *Morganella morganii* and *Corynebacterium aurimucosum*. The resistance pattern was available in one patient only as this is not routinely performed and only tested on request.

### Laboratory findings and imaging

Overall, pyuria was found in nine patients (45%) (Table 2). C-reactive protein (CRP) was measured in six patients and elevated (> 5 mg/L) in five. Kidney function was measured in five patients and reported as normal in all. Both patients with AFBN had an elevated CRP, nephritic foci in magnetic resonance imaging (MRI) and no other pathogen detection.

### Treatment

Overall, 14/20 patients (70%) were treated with antibiotics, including all patients with UTI or malodorous urine. Oral amoxicillin, amoxicillin/clavulanic acid or cefpodoxime or intravenous third-generation cephalosporins (ceftriaxone or ceftazidime) were used for treatment. Patients with AFBN were treated with third-generation cephalosporins for 3 weeks. Patients with malodorous urine were all treated with oral amoxicillin or amoxicillin/clavulanic acid for 5–7 days. Five patients required hospital admission, due to decreased general condition and/or the need for intravenous antibiotic treatment.

### Outcome

All patients with UTI were successfully treated with a clinical cure. However, the recurrence of *A. urinae* detection occurred in two patients, one after 2 months and one after 2 years. In patients with malodorous urine, the disappearance of the extraordinary unpleasant smell was achieved in all patients. One patient had recurrent episodes with malodourous urine and *A. urinae* detection until the surgical treatment of urethral valves.

## Discussion

Our study indicates that *A. urinae* in children is predominantly detected in boys. Malodorous urine and UTI with *A. urinae* were only seen in boys. Interestingly, this finding contrasts reports in adults, in whom *A. urinae* bacteriuria is found more often in females or at similar frequencies in both sexes [4, 15–17]. Invasive infections with *A. urinae* bacteraemia, however, are typically found in elderly men with urogenital disorders [18]. The reason for this variable sex distribution between the different age groups remains unclear.

In our study population, urogenital disorders were present in two-thirds of the patients, including all patients with a urinary tract infection. Patients with urogenital disorders were also more likely to have recurrent *A. urinae* infections. Our findings correlate with the literature in adults, which describes an association of urogenital disorders with *A. urinae* bacteriuria or infections [4, 18]. Urogenital disorders were also described in some of the case reports on *A. urinae* infections in children. A bladder diverticulum was found in a 5-year-old boy with malodorous urine [8], and prior pyeloplasty and VUR were described in a 12-year-old boy with an *A. urinae* UTI [10]. The association of urogenital disorders and recurrent detection also correlates with data from a mouse model that showed susceptibility to prolonged bacteriuria with *A. urinae* in mice with inherent vesicoureteral reflux [19]. Our findings together with the existing literature suggest that *A. urinae* bacteriuria may indicate an underlying urogenital disorder.

Mixed infections were documented in this case series, suggesting that *A. urinae* is part of the urogenital flora. Also, mixed cultures including *A. urinae* have been described to lead to malodorous urine [6]; however, this seems not always to be the case, as shown in one of our patients who had a malodorous urine without a mixed culture. It is well known, that pathogens produce different bacteria-specific volatile organic compounds that can cause a characteristic smell [20]. Physicians in our study described the malodorous urine as a foul or with a fishy smell. This smell has been attributed trimethylaminuria which can be produced by bacteria species such as *Pseudomonas* and *E. coli* [21] and likely also by *A. urinae*.

Our study is the first to show more severe presentations associated with the detection of *A. urinae* in several children. So far, only one previous case was described with an *A. urinae* infection causing a UTI in a 12-year-old boy [10]. In our study, we detected four cases of UTI with *A. urinae*, all of which occurred in boys and were associated with urogenital disorders. Two of these cases were diagnosed with AFBN. Whether *A. urinae* was the cause of the infection in these two children is debatable. There is an association between urogenital disorders and AFBN, which might lead to *A. urinae* being present as a bystander bacterium and not as an infective cause [14]. However, the detection of *A. urinae* monocultures in both cases with AFBN suggests there is a causal relationship. Bacteraemia and systemic infection with *A. urinae* were described in the literature including two case reports with endocarditis in the paediatric population [11, 12]. These findings suggest that invasive infections with *A. urinae* like AFBN are conceivable.

This study was limited by the number of patients and by its retrospective design. Unfortunately, informed consent could not be obtained for ten children, and therefore, these cases were not included. Despite its clinical significance in selected cases, *A. urinae* remains an unusual pathogen in the paediatric age group. Nevertheless, to the best of our knowledge, this is the largest case series describing the spectrum of *A. urinae* infection in the paediatric population. Our findings are likely transferable to other similar settings given its long observation period and should raise awareness for this unusual pathogen in the paediatric age group. We suggest further investigations of *A. urinae* in the context of urinary tract infections with a multi-centre study to provide further evidence. In addition, a prospective study would allow a standardized protocol to collect clinical and laboratory data and give more information about the long-term follow-up.

In conclusion, our findings show that *A. urinae* is not only causing malodorous urine but may lead to a more severe presentation. Our study suggests that in paediatric populations, *A. urinae* predominantly affects boys. As most cases were associated with urogenital disorders, a nephro-urological investigation should be considered when *A. urinae* is detected.

## Data Availability

Upon request relevant documentation and data can be presented for data transparency.

## Abbreviations

AFBN: Acute focal bacterial nephritis
AMC: Amoxicillin-clavulanic acid
AMX: Amoxicillin
*A. urinae*: *Aerococcus urinae*
BBD: Bladder bowel dysfunction
CAKUT: Congenital anomaly of the kidney and urinary tract
CRP: C-reactive protein
IQR: Interquartile range
MALDI-TOF MS: Matrix-assisted laser desorption ionization time of flight mass spectrometry
MRI: Magnetic resonance imaging
Ns: Not specified
PUV: Posterior urethral valves
UTI: Urinary tract infection
VUR: Vesicoureteral reflux
WBC: White blood cell
